# Shaping the Metabolism of Intestinal *Bacteroides* Population through Diet to Improve Human Health

**DOI:** 10.3389/fmicb.2017.00376

**Published:** 2017-03-07

**Authors:** David Rios-Covian, Nuria Salazar, Miguel Gueimonde, Clara G. de los Reyes-Gavilan

**Affiliations:** Department of Microbiology and Biochemistry of Dairy Products, Instituto de Productos Lácteos de Asturias, Consejo Superior de Investigaciones Científicas (IPLA-CSIC)Villaviciosa, Asturias, Spain

**Keywords:** *Bacteroides*, propionate, branched-chain amino acids, short chain fatty acids, diet, human metabolism, intestinal microbiota

## Intestinal microbiota and the control of glucose homeostasis and lipid metabolism in the host

The human intestinal microbiota is dominated by five phyla: Firmicutes, Bacteroidetes, Actinobacteria, Proteobacteria, and Verrucomicrobia. In adults, more than 80% of the species belong to just two phyla, Firmicutes and Bacteroidetes. Short chain fatty acids (SCFA) are catabolic end-products from intestinal microbial fermentation. Acetate, propionate and butyrate are the most abundant (Ríos-Covián et al., 2016a) whilst branched chain fatty acids (BCFA; isobutyrate, valerate, and isovalerate), the organic acids lactate, succinate, formate, and gases, can be also formed.

In humans, the main fermentable sources of SCFA are undigested dietary polysaccharides; amino acids and proteins may constitute additional substrates for colonic fermentation, whereas host-derived glycoproteins contribute to a limited extent. BCFA can be formed at considerably lower proportions than SCFA from branched-chain amino acids (BCAA; valine, leucine, and isoleucine). Threonine renders propionate and butyrate, whereas glutamate, histidine, lysine, arginine, and alanine give rise to acetate and butyrate formation; additionally, the intestinal microbiota contributes to the production of amino acids available to the host through *de novo* biosynthesis (Neis et al., 2015). Moreover, metabolic cross-feeding, that is the utilization of end products from the carbohydrate catabolism of a given microorganism by another one, strongly influences the final balance of intestinal SCFA. It occurs mainly for the formation of butyrate from acetate or lactate, is considerably lower for butyrate conversion to propionate, and very scarce between propionate and acetate (Den Besten et al., 2013).

Intestinal SCFA can incorporate into the enterohepatic circulation, being metabolized in the liver and reaching other extra-intestinal locations (Den Besten et al., 2014). Increasing evidence supports a regulatory role for SCFA in glucose homeostasis and lipid metabolism, in which intestinal SCFA ligands FFAR2 and FFAR3 and the glucagon-like peptide are involved. In the liver propionate is gluconeogenic whereas acetate and butyrate are lipogenic. Recent studies evidence that propionate and butyrate activate the intestinal gluconeogenesis (De Vadder et al., 2014), the glucose synthesized serving as a homeostatic signal in the portal system, to control hepatic gluconeogenesis (causal factor of insulin resistance and type 2 diabetes) and improving whole-body glucose homeostasis. Moreover, propionate flux through the liver reduces visceral and liver fat by decreasing intrahepatic triglycerides (Chambers et al., 2015). Propionate inhibits hepatic lipogenesis and cholesterogenesis promoted by acetate (Demigne et al., 1995) whereas propionate and butyrate inhibit lipolysis and lipogenesis and increase the incorporation of glucose mediated by insulin into the adipose tissue (Heimann et al., 2015). These observations prompt to the acetate/propionate ratio as an indicator for the potential contribution of intestinal SCFA to body lipid metabolism. Additionally, the improvement of glucose homeostasis promoted by dietary fiber seems to be associated with elevated fluxes of SCFA from the intestinal lumen to other organs rather than with the fecal SCFA concentrations (Den Besten et al., 2014).

Several metabolic disorders as obesity, insulin resistance, and metabolic syndrome are associated with impairment of the metabolism of carbohydrates and lipids by the host, and are accompanied by changes in the gut microbiota (Turnbaugh et al., 2009; Bervoets et al., 2013). Higher levels and altered patterns of SCFA (Fernandes et al., 2014; Salazar et al., 2015) and changes in the Firmicutes to Bacteroidetes ratio, have been repeatedly reported in obese individuals (Ley et al., 2006; Turnbaugh et al., 2006). Nonetheless, contradictory results published so far on the relative abundance of both phyla exclude its use as a broadly applicable marker.

Increases in plasma circulating BCAA and aromatic amino acids (phenylalanine and tyrosine) were related with higher risk of type 2 diabetes and insulin resistance (Utzschneider et al., 2016), having been suggested that the altered functionality of the intestinal microbiota (also affecting *de novo* biosynthesis of amino acids) determines these differential profiles of circulating amino acids (Neis et al., 2015).

Increasing protein intake (Pillot et al., 2009) and gastric surgery (Liou et al., 2013) have demonstrated efficacy for weight control and improvement of glucose homeostasis, partly related to the increase of propionate (De Vadder et al., 2014), and enrichment of intestinal Bacteroidetes/*Bacteroides* (Furet et al., 2010; Jumpertz et al., 2011; Liou et al., 2013). In contrast, a significant reduction in butyrate and certain butyrate-producing Firmicutes have been associated with diets containing low amounts of fiber and carbohydrates (Duncan et al., 2007, 2008; Walker et al., 2011). These suggest a microbiota unbalance in obese subjects, or under inadequate diets, which is partly restored following gastric surgery or by introducing weight-loss diets. However, some microbiome alterations seem to persist after dietary interventions, facilitating post-dieting weight regain (Thaiss et al., 2016). This stresses the importance of achieving a full restoration of the intestinal microbiota after dietary treatments, including proper balanced microbial metabolic products, to ensure durable effects.

## A focus on the genus *Bacteroides* and the production of propionate in the intestinal microbial ecosystem

The order Bacteroidales is the most abundant Gram-negative bacteria, colonizing the human gut at densities up to 5–8 × 10^10^ CFU per gram of feces (Zitomersky et al., 2011). Among the predominant genera are *Bacteroides* and *Prevotella*. These microorganisms can use a wide range of dietary soluble polysaccharides that are firstly released from vegetable fiber in the intestine by microbial primary degraders (Martens et al., 2011). The genus *Bacteroides* displays a high flexibility to adapt to the nutritional conditions of the intestinal environment (Comstock and Coyne, 2003), being able to use dietary or host-derived glycans according to the nutrient availability (Sonnenburg et al., 2005). *Bacteroides* can also incorporate amino acids from outside (Smith and MacFarlane, 1998) which could be used to maintain cell structures and as an energy source.

Three different biochemical pathways have been identified in colonic bacteria for propionate formation (Reichardt et al., 2014). The succinate pathway is the only one for propionate production from hexoses by Bacteroidetes, although some Negativicutes (family *Veillonellaceae*, phylum Firmicutes) can form propionate by utilizing succinate. The acrylate pathway is used for the conversion of lactate into propionate by very few bacterial genera within the phylum Firmicutes, whereas deoxy-sugars (fucose and rhamnose) are converted through the propanediol pathway by some Proteobacteria and members of the *Lachnospiraceae* family (phylum Firmicutes). *Akkermansia muciniphila* (phylum Verrucomicrobia) has been identified as a key propionate producing mucin-degrading species (Derrien et al., 2004).

Notably, several studies point to Bacteroidetes as the largest propionate producers in the human gut (Salonen et al., 2014; Aguirre et al., 2016). Interestingly, by modifying microbiota composition with antibiotic treatment in mice, Zhao et al. (2013) found a strong correlation between fecal levels of SCFA and the abundance of *Bacteroides* and other members of the phylum Bacteroidetes.

## The type of carbohydrates and availability of organic nitrogen sources modify *In vitro* the metabolism of *Bacteroides*

SCFA and organic acids formed in cultures of *Bacteroides* (acetate, succinate, lactate, and propionate) depend on the type of fermentable substrates, generation time and incubation period (Kotarski and Salyers, 1981; Rios-Covian et al., 2013, 2015, 2016b). Propionate is generally favored at long generation times, with complex carbohydrates, and under carbon source limitation.

**Figure 1:**
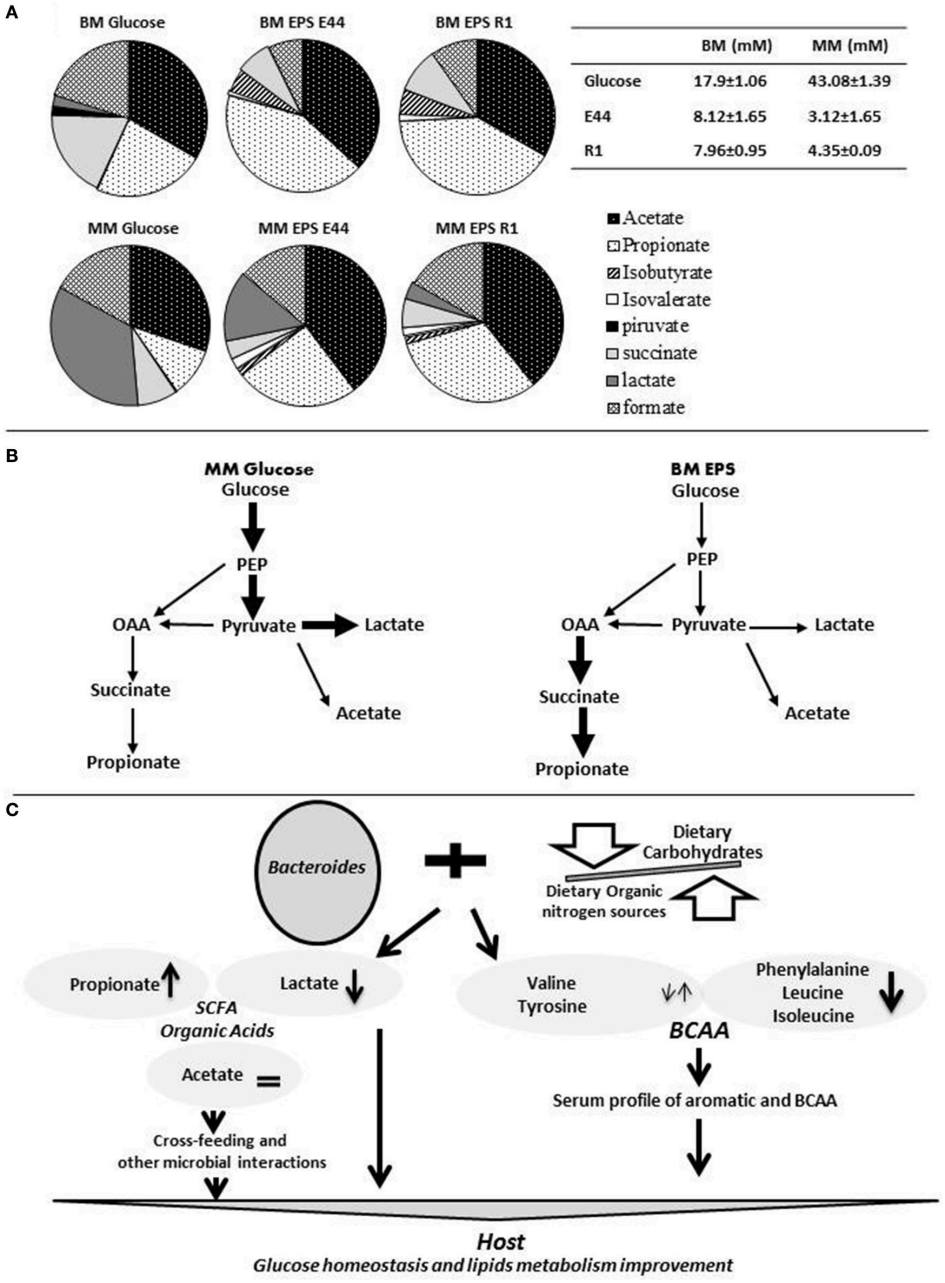
**The metabolic versatility of ***Bacteroides*** and the modulation of its metabolism through diet may impact human health. (A)** The relative proportions of the different organic acids and SCFA produced by cultures of *Bacteroides fragilis* at 24 h of incubation in non-defined peptone and yeast extract containing medium (BM; Rios-Covian et al., 2015) and in minimal medium without no organic nitrogen source (MM; Rios-Covian et al., 2016b) and supplemented with glucose, or with exopolysaccharides produced by Bifidobacterium strains (EPS E44 and EPS R1), are represented in shaded circles. The table at the top right side indicates total concentration (mM) of SCFA plus organic acids produced by B. fragilis in the different culture conditions. **(B)** Schematic representation of catabolic routes for the formation of SCFA and organic acids by *B. fragilis*. Thick bold arrows indicate pathways probably favored in MM supplemented with glucose (left side) or in BM supplemented with bacterial EPS (right side). **(C)** Schematic representation of the general hypothesis on how re-shaping the intestinal Bacteroides metabolism through the adequate balance of dietary proteins and carbohydrates could influence human health. On the one hand, changes occurring in the profile of SCFA and organic acids produced by this bacterium could act on the host carbohydrates and lipids metabolism directly or through cross-feeding or other microbial interaction mechanisms. On the other hand, the metabolism of intestinal Bacteroides may modify blood circulating amino acids in the host, which have been related with some metabolic disorders. OAA, oxaloacetate; SCFA, short chain fatty acidis; BCAA, branched chain amino acids; PEP, phosphoenolpyruvate.

We have studied the metabolism of *Bacteroides fragilis* growing in media containing different carbohydrates and nitrogen sources. Catabolic end-products formed in the presence of carbohydrates in non-defined peptone and yeast extract containing medium (BM; Rios-Covian et al., 2015) with respect to a minimal medium without no organic nitrogen source (MM; Rios-Covian et al., 2016b) evidenced higher SCFA and organic acids production in the former medium, when it was supplemented with bacterial exopolysaccharides (EPS), which are complex structures synthesized by some bacteria (Figure 1A). Acetate accounted for 30–54% of the total products formed in any condition, constituting a fundamental way for obtaining energy by this bacterium. An inverse correlation was found between the production of propionate plus succinate and that of lactate (Rios-Covian et al., 2015, 2016b; Figure 1A), this last being favored in the absence of organic nitrogen sources and with rapid fermentable carbohydrates, as occurs in MM added with glucose. Conversely, a shift toward propionate formation appears to occur in the presence of organic nitrogen when EPS are present. This probably reflects a preferential use of the glucolytic pathway and acetate formation for obtaining energy and keeping redox balance by *B. fragilis* in the presence of rapidly fermentable carbohydrates; in contrast, when complex/slowly fermentable carbohydrates are available and amino acids are present, carbon skeletons from amino acids could be incorporated at the level of pyruvate; in such conditions the propionate-succinate pathway seems to be potentiated as a way for energy obtaining whilst serving to restore cell redox balance (Rios-Covian et al., 2015; Figure 1B). Proteomics and gene expression analyses reinforced the hypothesis of the activation of amino acids catabolism and the succinate pathway in *B. fragilis* grown in BM with EPS (Rios-Covian et al., 2015). Therefore, the preferential metabolic route for energy production and redox maintaining, and the final metabolic products formed by *B. fragilis*, may be largely dependent on carbohydrates and nitrogen sources available. These results suggest the possibility of regulating the metabolism of *Bacteroides* by controlling dietary carbohydrate/protein balance.

Moreover, when analyzing the amino acids in cultures of *B. fragilis* added with different carbohydrates, we found a decrease in the concentration of leucine, isoleucine and phenylalanine after incubation in any condition, whereas valine and tyrosine showed much less increases or slight decreases in EPS as compared to glucose (Supplementary Material Table 2 in Rios-Covian et al., 2015). This points to a potential capacity of *B. fragilis* (as may probably occur with other Bacteroidetes) for regulating BCAA and aromatic amino acids levels in its growth environment.

## Modulation of the intestinal *Bacteroides* by dietary carbon/nitrogen sources: a tool for restoring the intestinal microbiota metabolic balance

Under sufficient organic nitrogen, the mildly acidic pH (5.5) stimulates butyrate producing species in the human colon curtailing the growth of *Bacteroides* that was however maximized at pH 6.5 (Walker et al., 2005). The pH in the caecum is about 5.7 but gradually increases to 6.7 in the rectum. Dietary fiber fermentation promotes a slight decrease of the luminal pH whereas high protein/amino acids fermentation, favored by low carbohydrate availability, causes slightly pH increases (Smith and MacFarlane, 1998). Interestingly, a recent study with mice demonstrated that diet-microbiome interactions are driven by the pattern of protein and carbohydrate intake (Holmes et al., 2017). Moreover, some experiments with gnotobiotic mice support shifts in *Bacteroides* metabolic functions as a response to dietary changes (McNulty et al., 2013; Wu et al., 2015).

The studies just commented support the interdependence between diet, gut microbiome and host metabolism, and allow to hypothesize that the combination of dietary organic nitrogen sources with appropriate carbohydrates may be used to modify the metabolic activity of colonic *Bacteroides* populations by modifying the profile of organic acids formed and enhancing propionate formation in some parts of the large intestine while promoting shifts toward healthier profiles of serum amino acids (Figure 1C).

Within this “scenario,” the potential role that the functional control and metabolic reprogramming of *Bacteroides* through diet may play in the regulation of the host metabolism deserves more attention. It is essential to decipher to what extent organic nitrogen sources and carbohydrates could affect the different species of prominent intestinal bacteria, such as the genus *Bacteroides*. An important question raised is whether changes in SCFA and organic acids profile induced by remodeling the metabolic activity of *Bacteroides* through adequate dietary interventions would influence other less nutritionally versatile gut beneficial microbes through the enhancement of cross-feeding or other microbial interaction mechanisms. Omics, including metabolomics/metabonomics, applied to the analysis of microbial cultures, animal models, and human samples are necessary for understanding host and microbiota metabolic remodeling as a response to dietary combinations of organic nitrogen/carbohydrates.

The potential to re-shape the metabolism of *Bacteroides* with specific combinations of dietary carbohydrates-proteins based on their composition, structure and availability in the gut, merits further study. The final aim should be designing diets based on nutrient components targeted at modulating the metabolism of *Bacteroides*, which may interact with other intestinal beneficial microbes, in order to restore the metabolic balance of the microbiota to promote durable host's health effects.

## Author contributions

DR, NS, MG, and Cd conceived the idea and designed the structure of the manuscript. All authors contributed significantly to the experimental data compared in the Figure 1A. Cd and DR drafted the manuscript and Figure. All authors have critically red, corrected, and approved the final version of the manuscript and agree with the opinions expressed here.

## Funding

The work of the research group in the matter of this article is being currently financed by projects AGL2013-43770-R from Plan Estatal de I+D+I (Spanish Ministry of Economy and Competitiveness, MINECO) and by Grant GRUPIN14-043 from Plan Regional de Investigación del Principado de Asturias, Spain. Both national and regional grants received cofounding from European Union FEDER funds. DR-C was the recipient of a predoctoral FPI fellowship and NS benefits from a Juan de la Cierva post-doctoral contract, both granted by MINECO.

### Conflict of interest statement

The authors declare that the research was conducted in the absence of any commercial or financial relationships that could be construed as a potential conflict of interest.
